# In Vitro Assessment of *Bacillus thuringiensis* Exopolysaccharides and Their Effects on Gut Microbiota from Ulcerative Colitis In Vitro

**DOI:** 10.3390/ijms26041692

**Published:** 2025-02-16

**Authors:** Zexin Gao, Jie Tang, Chuanchao Wu, Wenping Ding, Xianyi Wang, Yaohang Long, Yaping Wang, Hongmei Liu

**Affiliations:** 1 Engineering Research Center of Medical Biotechnology, School of Biology and Engineering, Guizhou Medical University, Guiyang 550025, China; gzxwh0616@163.com (Z.G.); tang100705@163.com (J.T.); dingwenping19@mails.ucas.ac.cn (W.D.); wangxianyi@gmc.edu.cn (X.W.); longyaohang@gmc.edu.cn (Y.L.); hnlywangyaping@163.com (Y.W.); 2 Key Laboratory of Carbohydrate Chemistry and Biotechnology, Ministry of Education, Jiangnan University, Wuxi 214122, China; wuchuanchaowcc@foxmail.com; 3 School of Basic Medicine Science, Guizhou Medical University, Guiyang 550031, China

**Keywords:** *Bacillus thuringiensis*, exopolysaccharide, functional properties, ulcerative colitis

## Abstract

*Bacillus thuringiensis* exopolysaccharide BPS-2 inhibits malondialdehyde secretion, enhances antioxidant enzyme activities, and significantly improves the antioxidant status of inflammatory cells. In the present study, the apparent morphology and spatial conformation of BPS-2 were analyzed further, and several functional properties were investigated. The results demonstrated that BPS-2 was a polymeric straight-chain polysaccharide with good thermal stability, exhibiting non-Newtonian properties and good antioxidant and anticancer activities. Notably, this study systematically investigated the impact of BPS-2 on the intestinal microbiota composition in patients diagnosed with ulcerative colitis. Through in vitro fermentation of fecal bacteria collected from six volunteers, it was found that BPS-2 exerted a positive influence on the intestinal flora of ulcerative colitis patients, augmenting the secretion of short-chain fatty acids and facilitating an increase in the relative abundance of *Bifidobacterium* spp. These results suggest that BPS-2 has the potential to be a food additive for suppressing ulcerative colitis and for other medically related applications.

## 1. Introduction

Bacterial exopolysaccharides (EPS) are macromolecules synthesized by bacteria and are of interest due to their diverse functional properties [1]. EPS are important for supporting the survival of microbial cells, including protection, aggregation, and interactions [2]. Currently, commercialized bacterial EPS include xanthan, thermogel, curdlan, and pullulan. They have the advantages of high adsorption, high biodegradability, and high biocompatibility, making them ideal for industrial applications [3,4,5]. Importantly, the composition of bacterial EPS is strictly dependent on their genes and cultural environment. *Bacillus* is an important genus of bacteria, characterized by tolerance to extreme environments and genetic stability for long-term preservation [6]. There are numerous reports of *B. thuringiensis* being used as a biopesticide [7,8,9]. To date, only one report outside the scope of biopesticide research has been published on the application of *B. thuringiensis* EPS [10].

Ulcerative colitis (UC) is a chronic, nonspecific inflammatory disease. Lesions occur primarily in the descending colon, sigmoid colon, and rectum and may spread further throughout the colon, with recurrence in severe cases [11]. To date, the etiology and pathogenesis of UC remain unclear because it is the result of a combination of factors, including oxidative damage, immune disorders, intestinal flora, and genetics [12]. The most frequently used drugs to treat UC disease are mesalazine, immunomodulators, and glucocorticoids. Because these drugs are relatively expensive, and UC is characterized by constant recurrence, the financial and psychological stress on patients is exacerbated [13]. Therefore, alternative substances with an acceptable safety profile need to be found for long-term suppression of UC [14]. It has been revealed that supplementation with glycosaminoglycans (GAGs) can repair the intestinal epithelial barrier and serve as an effective treatment for UC [15,16,17]. Common GAGs include hyaluronic acid, heparin, and chondroitin sulfate, which are mainly found in mammalian connective tissues. The absence of the defense barrier of GAGs in the host is one of the main causes of UC.

Studies have demonstrated that regulation of the intestinal flora has an essential role in alleviating UC, including maintaining the integrity of the intestinal barrier, enhancing immunity, and suppressing intestinal pathogens [18]. However, the effects of GAGs on intestinal microorganisms have been reported rarely. In addition, EPS has specific reactive groups, such as benzyl, acetyl, and phosphate groups, which can also significantly enhance antioxidant and anticancer activities [19]. In previous research, the immune-anti-inflammatory effect of BPS-2, a crucial EPS component of *B. thuringiensis* IX-01, upon the stimulation of RAW 264.7 cells was assessed [20]. The isolated BPS-2 was a heteropolysaccharide with a molecular weight of 29.36 kDa. The backbone of its basic unit was composed of four 1,4-β-GaINAc, four 1,4-β-GlcNAc, and two 1,4-α-Glc*p*, and the branched chain was composed of one 1,4-β-Man*p*, one T-β-Glc*p*, one 1,6-α-GaINAc, and two 1,5-α-Ara*f*, with 10 to 11 such basic units constituting BPS-2.

Based on the activity of BPS-2, as a derivative of GAGs, and considering the literature reports that GAGs have a repair function regarding the colonic barrier, it was hypothesized that BPS-2 also has a role in regulating the UC intestinal flora. Therefore, fecal flora was collected from UC volunteers to simulate fermentation in an in vitro colonic model, thereby exploring the effect of BPS-2 on human UC intestinal flora. In addition, the apparent morphology, thermal stability, rheological properties, and antioxidant and anticancer activity of BPS-2 were evaluated.

## 2. Results and Discussion

### 2.1. Surface Morphology Analysis of BPS-2

**Figure 1:**
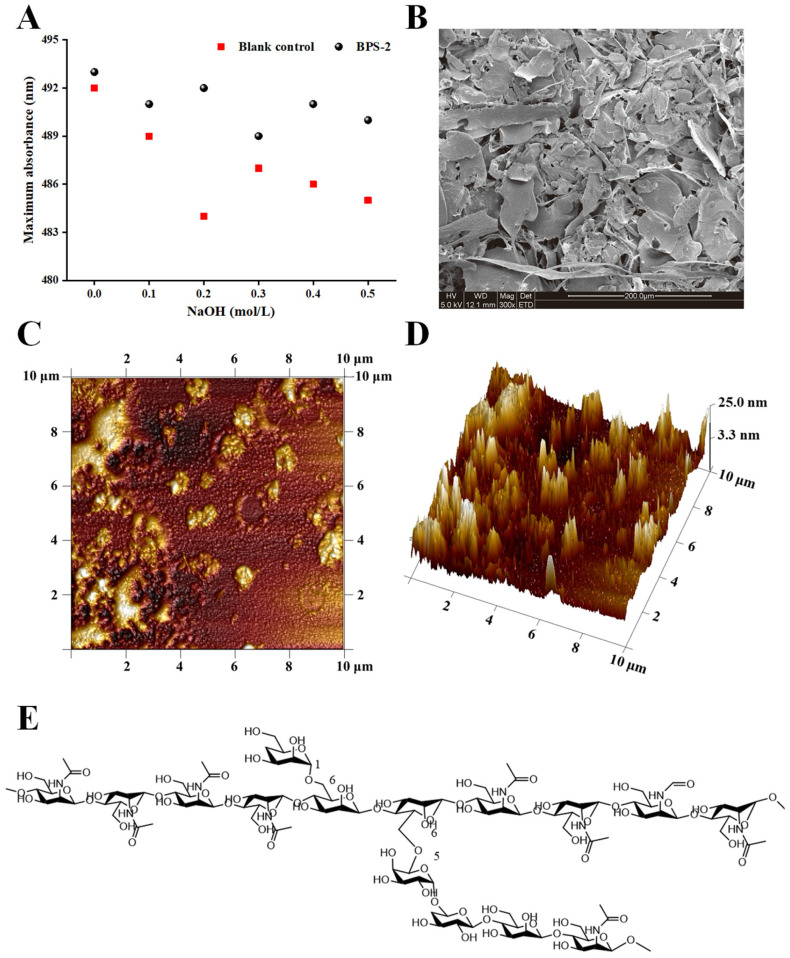
The results of the BPS-2 multimodal detection. Results of Congo red test (**A**), SEM micrographs (**B**), AFM microscope plain image (**C**), AFM microscope stereo image (**D**), and structure of the base unit of BPS-2 (**E**).

Congo red is an acid dye that forms complexes with polysaccharides in a three-strand helical conformation. As indicated by the Congo red test (Figure 1A), both the EPS part of *B. thuringiensis* BPS-2 and the blank control decreased to the same extent with an increase in NaOH concentration in the range of 0 to 0.5 mol/L, thus indicating that BPS-2 does not have a three-strand helical structure.

A scanning electron microscope (SEM) was used to observe the composition and morphology of the surface of ultra-structures. As shown in the SEM images (Figure 1B), the surface morphology of BPS-2 is composed of many sheets of varying morphology, stacked together tightly and irregularly, but the sheets still have three-dimensional pores of varying sizes between them, which can provide the material with the capacity to swell and retain water [21].

In recent years, atomic force microscopy (AFM) has been used widely to observe the physical morphology and macromolecular chain size of polysaccharides. In the AFM plain image (Figure 1C), different degrees of aggregation (0–15.91 nm in diameter) were observed between the BPS-2 molecules. Given that the BPS-2 polysaccharide was originally known to have a structure with two branched chains, a variable degree of aggregation likely resulted from the combined action of the branched and straight chains. As shown in the AFM stereogram (Figure 1D), the molecule of BPS-2 demonstrated a punctate or peak-like structure with a height ranging from 0.92 to 39.46 nm; by contrast, other studies have demonstrated that the usual single chain height of polysaccharide molecules is around 0.1~1.0 nm [22], which indicates that the polysaccharide mono-chain of BPS-2, as a base unit, is able to cross-link with multiple mono-chains. The reason for this intermolecular cross-linking of polysaccharide is due to the interaction of the branched chains with the non-carbon substituents on the polysaccharide through the negative and positive charges on the sugar chain [23].

Combining the known chemical structure of BPS-2 base unit (Figure 1E) and the lack of three-strand helix structure, it could be surmised that the spatial conformation of BPS-2 was mainly tandem with straight chains; however, when the polysaccharide concentration was high, its branched chains would join the long chains formed by their respective tandem to form a lamellar macromolecular substance, and this phenomenon was consistent with Figure 1B.

### 2.2. Functional Features of BPS-2

**Figure 2:**
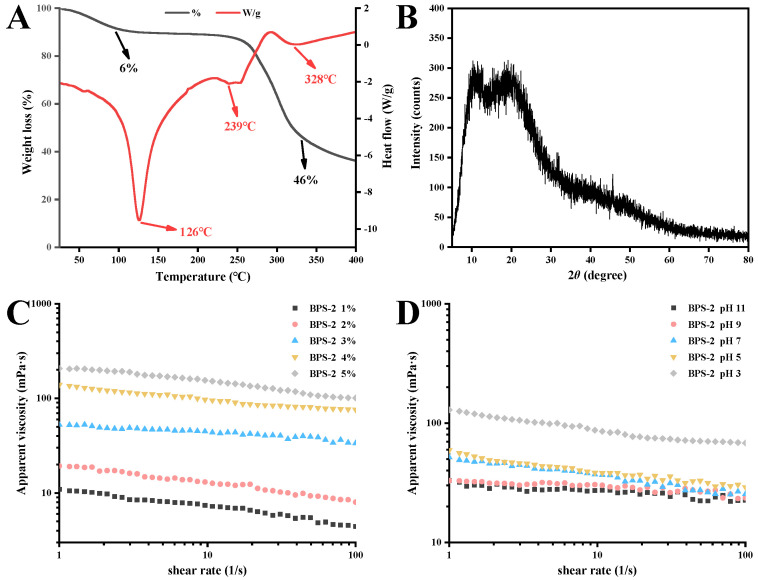
The BPS-2 thermal, crystallographic, and rheological properties. TG and DSC curves of BPS-2 (**A**), XRD of BPS-2 (**B**), effect of shear rate on the apparent viscosity of BPS-2 at different concentrations (**C**), and effect of shear rate on the apparent viscosity of BPS-2 at different pH levels (**D**).

The understanding of the thermal properties of polysaccharides is important in the industry. As shown in Figure 2A, the thermogravimetric (TG) and differential scanning calorimetry (DSC) curves of BPS-2 powder varied with increasing temperature. In the DSC curve, BPS-2 showed a strong endothermic transition peak at 126 °C, which represents the loss of water from the polysaccharide [24]. Two small endothermic peaks were exhibited at 239 and 328 °C, representing the thermal depolymerization of polysaccharides and the breakage of polysaccharide glycosidic bond linkages, respectively [25]. Interestingly, only two stages of mass loss were observed in the TG curves, describing a mass loss of BPS-2 from dehydration between 70 and 150 °C (6%) and a mass loss of up to 46% due to the decomposition of functional groups between 240 and 350 °C. Below 250 °C, the mass loss of BPS-2 was minimal (13%), but the total mass loss after thermal degradation at 400 °C was about 64%. The reason for the massive degradation of BPS-2 above 250 °C is presumed to be due to its mostly straight-chain polysaccharide structure. Some reports suggest that a highly branched polysaccharide structure can effectively increase thermal stability [26]. In summary, BPS-2 exhibits good thermal properties below 250 °C and can be processed at high temperatures to improve the material thermal stability.

To further clarify the crystal structure of BPS-2, its X-ray diffraction (XRD) spectrum was analyzed. The results show that BPS-2 has two clear diffraction peaks, indicating the presence of two crystal structures (Figure 2B). The diffraction peak of BPS-2 at around 2θ = 10° indicated a type I hydrated crystal structure, and the strongest diffraction peak at around 2θ = 20° corresponded to the type II crystal structure [27]. The type II crystal structure in the BPS-2 molecule is likely due to the monosaccharides with acetyl groups on their side chains, whereas the corresponding type I hydrated crystal structure originates from the monosaccharides with acetyl groups on the main chain [28].

Rheological analysis was performed to investigate whether BPS-2 was viscous, with the viscosity of the polysaccharides positively correlated with the concentration [29]. As shown in Figure 2C, the apparent viscosity of the BPS-2 solution increased with increasing concentration (1–5% *w*/*v*) and decreased with increasing shear rate, exhibiting non-Newtonian behavior. In the food industry, it is important to ensure the stability of polysaccharides under different pH conditions that exist in the stomach and small intestine. The apparent viscosity of BPS-2 solution gradually increased with decreasing pH and decreased with increasing shear rate (Figure 2D). However, the apparent viscosity of the BPS-2 solution at pH 3 was much higher than that of the other solutions, presumably because the acidic pH caused the acetyl groups on the straight chains of BPS-2 to be shed, resulting in the exposure of the polysaccharide hydrogen bonds to the coagulation effect, thus greatly increasing the apparent viscosity.

### 2.3. Antioxidant Capacity of BPS-2

**Figure 3:**
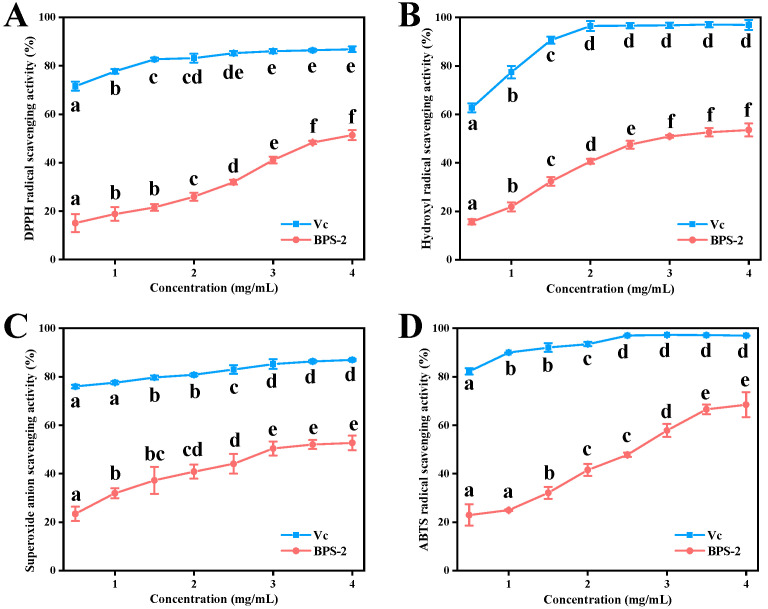
The antioxidant activity of BPS-2 at different concentrations. The scavenging activity regarding DPPH radicals (**A**), hydroxyl radicals (**B**), superoxide anions (**C**), and ABTS radicals (**D**). Vc refers to ascorbic acid. Data represent mean ± S.D. (*n* = 3). Significant differences between doses within the same experimental group are indicated by different letters after Dunnett’s test (*p* < 0.05).

The dose-dependent antioxidant behavior of BPS-2, as evaluated through four distinct radical scavenging assays, demonstrates its broad-spectrum redox modulation potential (Figure 3). The IC_50_ values followed a hierarchical order: 2,2′-azino-bis (3-ethylbenzothiazoline-6-sulfonic acid) (ABTS) < hydroxyl < superoxide anion < 1,1-diphenyl-2-picrylhydrazyl (DPPH), providing critical insights into its mechanism. Notably, BPS-2 exhibited the highest radical scavenging capacity against ABTS (IC_50_ = 2.62 mg/mL), likely due to its enhanced electron-transfer efficiency toward cationic radicals. In contrast, its relatively lower efficacy in neutralizing DPPH radicals (IC_50_ = 3.81 mg/mL) may be attributed to steric hindrance effects, which could limit its ability to donate hydrogen atoms to bulkier neutral radicals. This differential antioxidant performance is consistent with previous reports, indicating that aminopolysaccharides exhibit varying radical scavenging efficacy depending on the redox potentials and molecular dimensions of the target radical species [20].

Based on Duncan’s analysis, pairwise comparisons of the antioxidant data of Vc and each BPS-2 dosage group were made (Figure 3). In DPPH radical scavenging, Vc’s ability grew with concentration until 3 mg/mL (*p* < 0.05), while BPS-2’s became non-significant at 3.5 mg/mL (*p* < 0.05) (Figure 3A). For hydroxyl radical scavenging, Vc peaked at 2 mg/mL (*p* < 0.05), and BPS-2’s growth leveled off at 3 mg/mL (*p* < 0.05) (Figure 3B). BPS-2’s superoxide anion radical scavenging was similar to its hydroxyl radical scavenging, yet Vc was still superior (Figure 3C). In ABTS radical scavenging, BPS-2’s rate increased significantly (*p* < 0.05) to 68.49%, higher than the others. Vc’s peaked at 97.24% with non-significant growth from 2.5 mg/mL (*p* < 0.05) (Figure 3D). Notably, when benchmarked against natural antioxidants from other biological sources, BPS-2 demonstrates competitive activity. The observed similarity to *Mortierella alpina* polysaccharides reinforces the emerging paradigm that fungal-derived aminoglycans represent an underutilized reservoir of bioactive antioxidants [30]. The structural basis of BPS-2’s activity warrants particular attention. While the sixty-three percent amino-glycan content (D-GalN/GlcN) provides abundant nucleophilic amine groups for radical stabilization, recent studies suggest that the spatial distribution of these functional groups may be equally critical [31]. The observed bioactivity could arise from a unique three-dimensional configuration enabling simultaneous radical neutralization through multiple mechanisms: (1) hydrogen atom transfer via hydroxyl groups; (2) single electron transfer through amine moieties; and (3) radical stabilization via conjugated π–system interactions in the polysaccharide backbone [4].

The practical implications of these findings extend to functional food development and biomedical applications. The relatively low IC_50_ values in physiologically relevant radical models (particularly hydroxyl radicals and superoxide anions) position BPS-2 as a potential therapeutic agent for oxidative-stress-related pathologies.

### 2.4. Anticancer Activity of BPS-2

**Figure 4:**
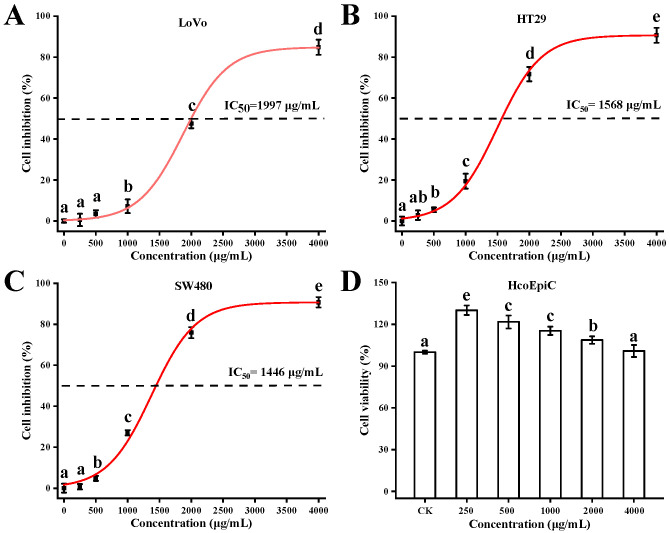
The results by MTT assay for anticancer effect of BPS-2 on different cell lines and cell viability. Anticancer effect of BPS-2 by MTT assay in LoVo (**A**), HT29 (**B**), and SW480 (**C**) cell lines. (**D**) The viability of HcoEpiC cell line treated with different concentrations of BPS-2 was also determined by the MTT method. CK was the control group. Data are expressed as mean ± S.D. of six independent experiments. Significant differences between doses within the same experimental group are indicated by different letters after Dunnett’s test (*p* < 0.05).

**Figure 5:**
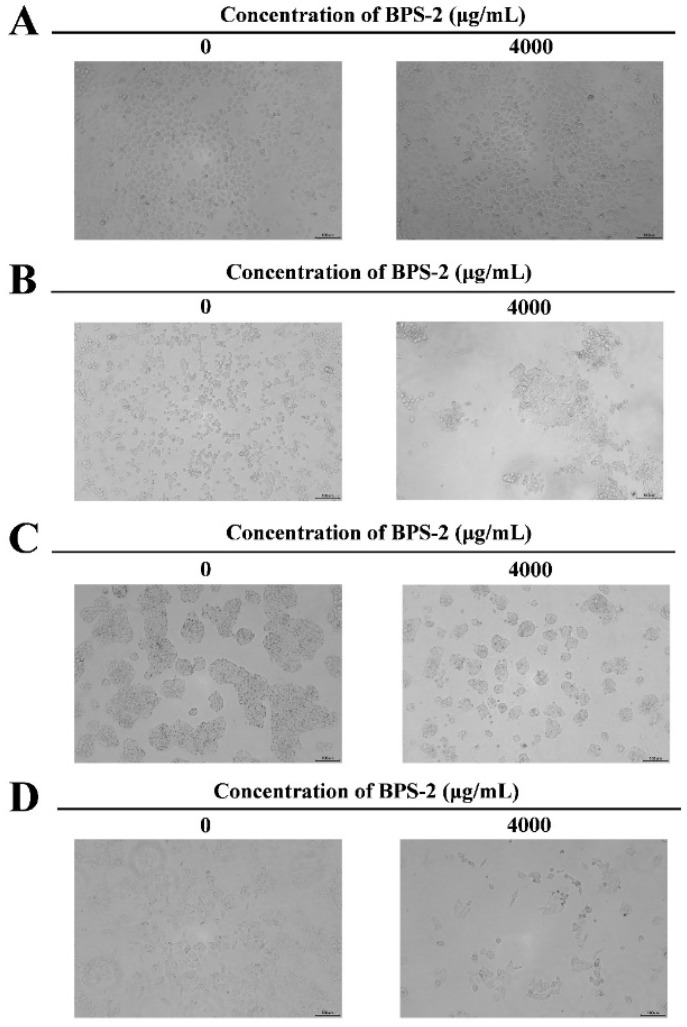
Toxicity test of BPS-2-fed cells. (**A**) HcoEpiC cells. (**B**) LoVo cell line. (**C**) HT29 cell line. (**D**) SW480 cell line. Scale bar = 100 μm.

The pronounced cytotoxic selectivity of BPS-2 toward colorectal cancer (CRC) cells (LoVo, HT29, and SW480) over human normal colorectal epithelial cells (HcoEpiC) highlights its potential as a tumor-selective therapeutic agent (Figure 4). The dose-dependent growth inhibition and morphological alterations (cell shrinkage/crumpling) observed at 4000 μg/mL suggest BPS-2 disrupts cancer cell proliferation via mechanisms distinct from conventional chemotherapeutics (Figure 5) [32].

Notably, the IC_50_ values (1446–1997 μg/mL) significantly surpass the activity of many reported polysaccharides (e.g., Mamani et al.’s chitin derivatives) (Figure 4A–C), likely due to BPS-2’s unique structural features [30]. Its high aminosaccharide content may enhance electrostatic interactions with negatively charged cancer cell membranes, facilitating receptor-mediated internalization or membrane destabilization. This aligns with studies demonstrating that polycationic polysaccharides induce apoptosis by activating caspase pathways or disrupting mitochondrial membrane potentials [17].

Based on the MTT assay to evaluate the anti-proliferative effect of BPS-2 on CRC cell lines (Figure 4A–C), the results show a significant association between drug concentration and cell-type-specific responses. At a concentration of 250 μg/mL, BPS-2 exhibited a statistically significant growth-inhibitory effect on HT29 cells (*p* < 0.05). Notably, when the concentration was increased to 1000 μg/mL, a significantly enhanced concentration-dependent effect was observed: the proliferation inhibition rate of SW480 cells reached 27.00%, which was significantly higher than 19.46% of HT29 cells (Δ7.54%) and 7.24% of LoVo cells (Δ19.76%). Thus, a cell-line-sensitivity gradient was established as SW480 > HT29 > LoVo. This dose-dependent differential sensitivity pattern suggests that the response mechanism of CRC cells to BPS-2 may involve cell-specific molecular characteristics. It is speculated that the heterogeneous expression of lectin-binding protein receptors among different cell lines, or differences in cell-surface glycosylation patterns that affect drug uptake, may be the key molecular basis for this phenomenon [32]. SW480′s lower IC_50_ (1446 μg/mL) could correlate with its aggressive phenotype, suggesting BPS-2 targets pathways critical for metastatic progression (Figure 4C). However, the high IC_50_ values (> 1000 μg/mL) underscore the need for structural optimization to improve potency [33]. While in vitro results are promising, the translational relevance requires validation in physiologically complex models. HcoEpiC at tested concentrations reduces concerns about gastrointestinal toxicity, but systemic effects must be evaluated in vivo. Future studies should investigate BPS-2’s pharmacokinetics, immune modulation, and synergy with standard therapies [17].

In conclusion, BPS-2 represents a structurally novel anti-CRC candidate with tumor-selective cytotoxicity. Its efficacy, coupled with low normal cell toxicity, justifies advanced preclinical development, prioritizing formulation strategies to enhance bioavailability and mechanistic studies to unlock its full therapeutic potential.

### 2.5. Effect of BPS-2 on Short-Chain Fatty Acids (SCFAs)

**Figure 6:**
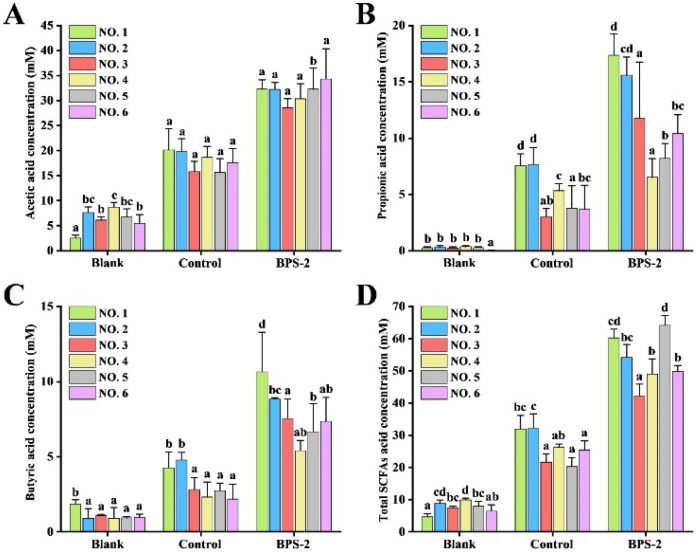
The changes in the concentration of SCFAs [acetic acid (**A**), propionic acid (**B**), butyric acid (**C**), and total acids (**D**)] after bacterial fermentation of feces from different volunteers supplemented with different carbon sources in BGR. The numbering of the volunteers in the figure matches the numbering in Table 1. The blank group represents the initial content of SCFAs in fecal bacteria of different volunteers. The control and BPS-2 groups represent the SCFAs content in fecal bacteria of different volunteers after 48 h of fermentation in BGR using starch and BPS-2 polysaccharide, respectively. Data are expressed as mean ± S.D. of three independent experiments. Different letters indicate significant differences among volunteers in the same treatment group by Dunnett’s test (*p* < 0.05).

SCFAs, particularly acetate, propionate, and butyrate, are important metabolites of the intestinal microflora and play a key role in maintaining several physiological functions of the host [34]. It has been demonstrated previously that BPS-2 is highly resistant to artificial saliva, gastric juice, and small intestinal fluid, with no significant changes in polysaccharide molecules; hence, BPS-2 reaches the colon without difficulty [20]. In this study, fecal bacteria from two healthy individuals and four UC patients were used for in vitro fermentation in a bionic gastrointestinal reactor (BGR) using the BPS-2 treatment and the control (starch); the concentration of SCFAs was measured after 48 h (Figure 6).

After 48 h, the concentrations of total SCFAs produced by fermentation of BPS-2 by intestinal bacteria in different volunteers (NO. 1–6) were 60.29 ± 2.68, 54.25 ± 3.91, 42.31 ± 3.62, 49.06 ± 4.73, 64.15 ± 3.01, and 49.84 ± 1.83 mM (Figure 6D); these values were significantly higher than the blank and the control group. The reason for the lower concentration of SCFAs in the blank group was that the intestinal barrier absorbs SCFAs. The fermentation of BPS-2 and the control group (starch) in vitro by BGR reflected the difference in the capacity of intestinal bacteria to convert different polysaccharide substances into SCFAs.

Rice is one of the staple foods of East and Southeast Asians, and starch is the main component of rice grain. In the control group, starch was selected as the reference compound. After 48 h of fermentation, two healthy individuals produced significantly (*p* < 0.05) higher levels of propionic acid, butyric acid, and total SCFAs than the four UC patients (Figure 6B–D). It indicated that intestinal bacteria were less capable of using starch to produce SCFAs in UC patients than healthy individuals.

Propionic acid reduces obesity by modulating immune cells in the host to achieve lower levels of fatty acids in the liver and plasma [34]. Butyric acid is an important energy supplier for colon cells and plays a key role in regulating the proliferation and differentiation of the colon epithelial cells, and it induces apoptosis in the colon cancer cells [35]. Interestingly, after 48 h of fermentation, UC patients in the BPS-2 group (NO. 3–6) indicated a significant increase in total SCFAs, with patient (NO. 5) having a significantly (*p* < 0.05) higher level of total SCFAs relative to the others (Figure 6D).

In summary, BPS-2 promotes the production of SCFAs, especially propionic acid and butyric acid, by intestinal bacteria in the healthy subjects and the ulcerative colon patients.

### 2.6. Intestinal Bacterial Diversity Analysis

**Figure 7:**
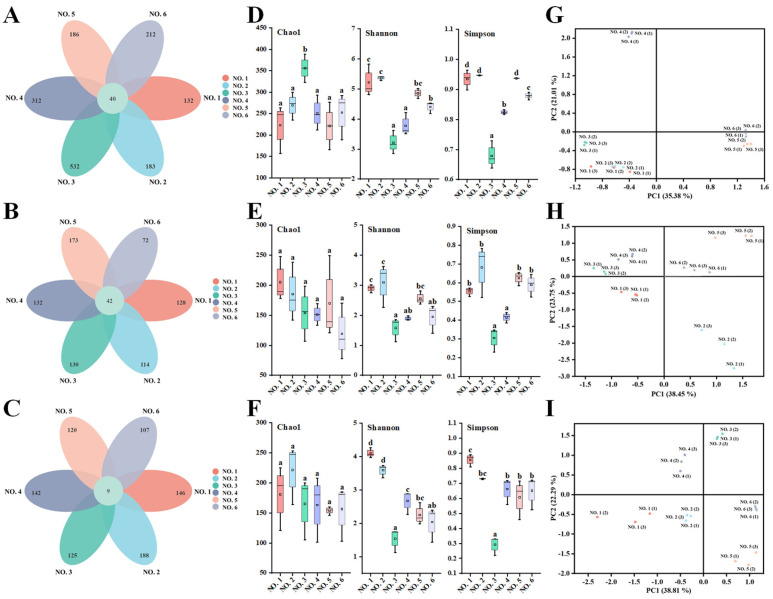
The α-diversity and β-diversity of fecal flora of different volunteers. Venn diagram of the initial fecal bacterial OTU levels of different volunteers (**A**) and after fermentation with starch (**B**) and BPS-2 (**C**). The Chao1, Shannon, and Simpson indices of the initial gut microbes of different volunteers (**D**) and after fermentation using starch (**E**) and BPS-2 (**F**). PCoA score plot based on weighted unifrac metrics for initial gut bacteria of different volunteers (**G**) and after fermentation using starch (**H**) and BPS-2 (**I**). The numbering of the volunteers in the figure matches the numbering in Table 1. Different letters represent significant differences (*p* < 0.05) among samples.

As shown in Figure 7A–C, 40 operational taxonomic units (OTUs) were shared among the initial gut bacteria of different volunteers, whereas 42 and 9 OTUs were shared when starch and BPS-2 were the main sources of ingested carbon, respectively. This indicates that when BPS-2 was used as the main carbon source, the abundance of the same species of gut bacteria significantly decreased in different volunteers, and the change in the species of gut bacteria was significantly (*p* < 0.05) higher than in the initial and starch groups. It is worth mentioning that the OTUs of the four UC patients in the initial group were significantly (*p* < 0.05) higher than the OTUs of the two healthy individuals, but the OTUs converged to the same level after in vitro fermentation was performed separately. It is possible that this phenomenon was caused by the higher abundance of harmful bacteria in the intestinal bacteria of UC patients compared with that of healthy individuals, and that in vitro fermentation was transferring harmful bacteria from an otherwise suitable environment for growth to an unfavorable environment, resulting in significant death of harmful bacteria.

The initial, fermented starch and BPS-2 gut bacterial α-diversity values of different volunteers are shown in Figure 7D–F. No significant differences were found in the intestinal bacteria Chao1 index of volunteers in different environments, except for the volunteer NO. 3 who had a more significant (*p* < 0.05) Chao1 index at the beginning, indicating that individual differences among people still exist. In addition, the Simpson and Shannon indices of initial gut bacteria and after fermented starch and BPS-2 treatments showed almost identical trends across the volunteers, with the two healthy ones being more significant on both indices than the four ulcerative colon patients, suggesting that the diversity of human gut bacteria is better in healthy individuals than in ulcerative colon patients. The flora composition (assessed by PCoA analysis) was similar in the two healthy individuals (Figure 7G–I), whereas the four UC patients had significantly different flora. Overall, the use of BPS-2 as the primary carbon source did not result in a reduction in the diversity of gut microbiota relative to starch as the control, which is normal for in vitro fermentation of the intestinal flora relative to the initial reduction.

### 2.7. Composition of the Intestinal Community

**Figure 8:**
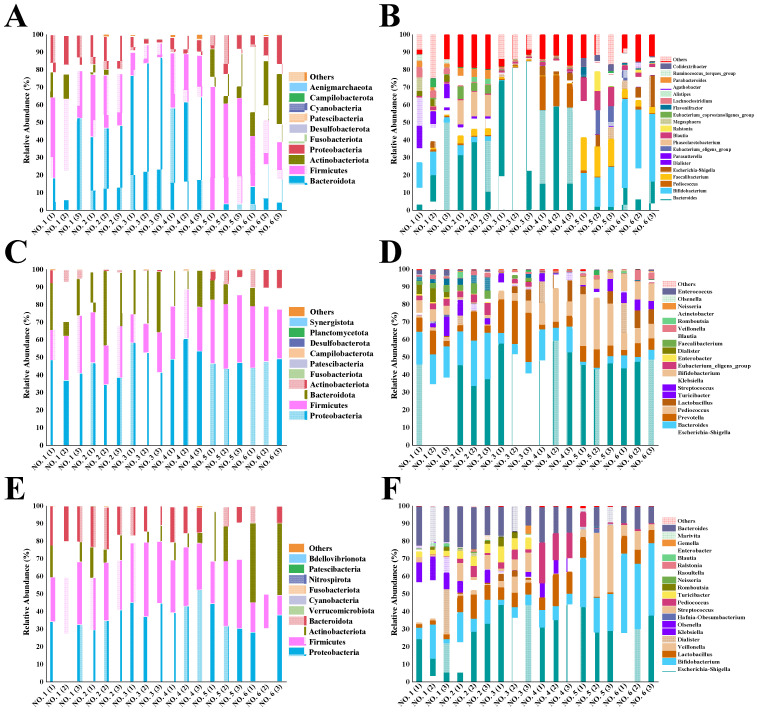
Changes in the relative abundance of phyla (**A**,**C**,**E**) and genera (**B**,**D**,**F**) in volunteer intestinal flora initially (**A**,**B**) and after fermentation with starch (**C**,**D**) and BPS-2 (**E**,**F**) as carbon sources. The experiments were replicated three times. The numbering of the volunteers in the figure is consistent with the numbering in Table 1.

In the natural world, 52 bacterial phyla are known to exist, but only 5–7 bacterial phyla are detectable in the mammalian gastrointestinal tract. The huge microbial community provides a mutually beneficial and functional treasure trove for the host, contributing to energy-harvesting channels and maintaining the normal functioning of the immune system and the production of essential vitamins; however, the flora can also have a detrimental effect in case of disease [36]. To reveal the specific composition of the initial gut microbial community in different volunteers, microbial differences were assessed at various taxonomic levels. At the phylum level (Figure 8A), four dominant bacterial phyla were identified in the six volunteers. Among them, Firmicutes, Bacteroidetes, Actinobacteria, and Proteobacteria had a combined relative abundance of more than 90%. Usually, the percentage of Firmicutes in the intestinal flora shows a negative relationship with gastrointestinal inflammation, whereas a significant increase in Proteobacteria and Bacteroidetes and a significant decrease in Firmicutes are found in UC patients [37].

Figure 8B shows the genera of the initial gut flora of the six volunteers. Volunteers NO. 1 and NO. 2, as healthy individuals, had more balanced intestinal flora. The initial flora of ulcerative colon patients (NO. 3–6) differed from that of the two healthy individuals. The relative abundance of *Bacteroides* in ulcerative colon patients NO. 3 and NO. 4 was as high as 80% and 58%, respectively, and the relative abundance of Firmicutes was only 10% and 26%. This phenomenon was detrimental, as the disruption of intestinal flora of UC patients was accompanied by a decrease in the ratio of Firmicutes to Bacteroidetes [38]. Interestingly, *Bifidobacterium* was detected in two UC patients (NO. 5 and NO. 6) with a relative abundance of 20% and 40%, respectively, which are high percentages, even though both patients indicated they had not ingested probiotics within a month (Table 1); thus, *Bifidobacterium* in these two individuals might have been ingested and established in their gut flora over 1 month prior. However, UC patients NO. 5 and NO. 6 showed the relatively low abundance of Bacteroidetes at 2% and 20%, respectively; it should be kept in mind that an imbalance in the relative abundance of either Bacteroidetes or Firmicutes in the intestinal flora is an important indication of adverse health effects.

**Figure 9:**
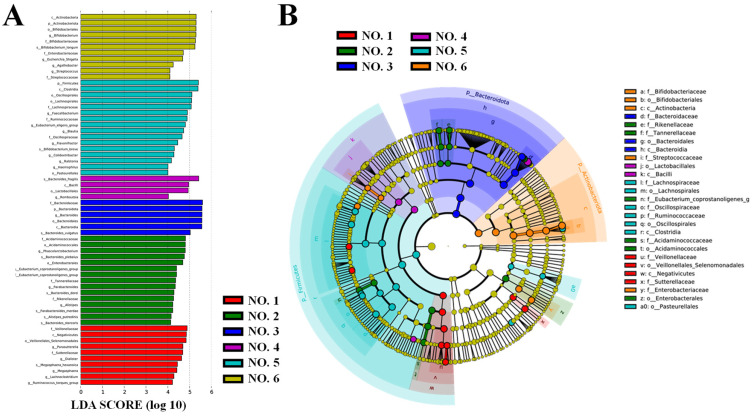
Taxonomic differences in the initial gut flora of different volunteers determined by LEfSe analysis (LDA score > 4.0). The LDA score distribution is histogram (**A**) and cladogram (**B**).

LefSe analysis was performed to identify statistically significant biomarkers of gut microbiota in the initial gut flora of the volunteers (Figure 9). As shown in Figure 9A, 62 taxa were found to differ significantly (*p* < 0.05) between groups at the phylum, order, family, genus, and species levels in the initial gut flora of different volunteers. Significantly enriched intestinal flora are indicated by the corresponding nodes, and their taxonomic levels are shown by line discriminant analysis (LDA) score distribution cladogram in Figure 9B. In the healthy volunteer NO. 1, *Parasutterella* was enriched significantly; it has a role in maintaining bile acid stability and coordinating cholesterol metabolism in the host [18]. By contrast, significant enrichment of *Bacteroides fragilis* and *Escherichia-Shigella* was identified in UC patients NO. 4 and NO. 6, respectively, as the hallmark flora that may cause inflammation [39]. In UC patient NO. 5, a significant enrichment of Clostridia was observed. Clostridia is a symbiotic genus in the intestinal tract of humans, but its proliferation can cause intestinal diseases, such as diarrhea, short-chain fatty acid metabolism disorders, stimulation of the immune system, inflammation, and disturbance of intestinal immune homeostasis due to the overgrowth of *Clostridioides difficile* [36]. Interestingly, in UC patient NO. 3, there was a high relative abundance of *Bacteroides vulgatus*, and it has been reported that the severity of UC is closely related to the proteases produced by *Bacteroides vulgatus* [40].

As shown in Figure 8C, the relative abundance of Proteobacteria increased in different volunteers after 48 h of fermentation in BGR. The reason for the increased abundance of Proteobacteria could be attributed to their likely reliance on polysaccharides with high glucose content for reproduction; straight-chain starch is one such polysaccharide. Among human intestinal microorganisms, Proteobacteria include mainly *Shigella*, *Salmonella*, and *Escherichia*, and these genera may be associated with intestinal inflammatory diseases. Therefore, reducing the abundance of the above-mentioned genera is beneficial to the health of the host [41]. In comparison, the relative abundance of Proteobacteria increased less with the intake of BPS-2 than starch (Figure 8E).

The relative abundance of Actinobacteria in the intestinal flora of each volunteer increased relative to the other groups after BPS-2 intake, suggesting that BPS-2 facilitated an increase in the relative abundance of Actinobacteria. The molecular weight of polysaccharides, the composition and ratio of monosaccharides, the type of glycosidic bonds and connections, the position and length of branched chains, and the spatial structure all have an important influence on the biological activity of polysaccharides [4]. Yu et al. [42] suggested that the composition and proportion of monosaccharides are also important structural features for the probiotic activity of polysaccharides. It was reported that EPS consisting of glucose, galactose, xylose, glucosamine, galactosamine, and mannose had the effect of increasing the abundance of probiotic flora and mitigating UC [43], and the composition of BPS-2 was similar to that of the reported EPS.

Considering the genera shown in Figure 8F, the main increase in the relative abundance of Actinobacteria was due to the contribution of *Bifidobacterium*. *Bifidobacterium*, a probiotic associated with human health, is closely related to the production of butyric acid, as well as having the function of enhancing the intestinal mucosal barrier and reducing the level of inflammation [12]. Notably, the intake of BPS-2 resulted in a more balanced relative abundance of intestinal flora in the six volunteers relative to the intake of starch (Figure 8D,F). Among the UC patients, the microbial community composition of UC patient NO. 3 was already similar to the two healthy individuals (NO. 1 and NO. 2), suggesting BPS-2 improved the colonic flora of UC patient NO. 3. In addition, macromolecular polysaccharides could potentially have a higher safety profile, lower toxicity, more pronounced prebiotic activity (especially for UC disease), as well as undergo continuous degradation during fermentation in the gut, leading to a more balanced production of SCFAs due to a mild fermentative effect produced by the complex structure [44]. Interestingly, after BPS-2 intervention, the increase in *Bifidobacterium* abundance was significantly higher (*p* < 0.05) in UC volunteers relative to the non-significant increase in healthy volunteers (Figure 8F). The abundance of bifidobacteria was found to be significantly and positively correlated with the concentration of monosaccharides Ara*f*, GalNAc, GlcNAc, and Man*p* in polysaccharides, but more research is needed due to the individual differences among different volunteers [45]. In summary, the capacity of BPS-2 to promote the production of SCFAs by the UC gut flora suggests that BPS-2 has the potential to improve gut health in UC patients.

## 3. Materials and Methods

### 3.1. Materials and Reagents

EPS-rich *B. thuringiensis* IX-01 was isolated from Pixian pea sauce from Sichuan, China. The strain was in China Centre for Type Culture Collection (CCTCC M 2020486), and the 16S rRNA sequence was deposited in NCBI with the registration number OL687443. The detailed method for the preparation of BPS-2 from IX-01 was described in a previous report [20]. Congo red reagent, SCFAs standards, and 3-(4,5-dimethylthiazol-2-yl)-2,5-diphenyltetrazolium bromide (MTT) were purchased from Sigma-Aldrich (St. Louis, MO, USA). All other chemical reagents were purchased from Sinopharm Chemical Reagent Co., Ltd. (Shanghai, China).

### 3.2. Congo Red Test

The three-helical structure of BPS-2 was probed by the Congo red test according to Xu et al. [46]. A BPS-2 sample (5 mg) was supplemented with 2.0 mL of distilled water and 2.0 mL of 0.08 mol/L Congo red reagent, and then different volumes of 1.0 mol/L NaOH solution were added to bring the NaOH solutions to concentrations 0, 0.1, 0.2, 0.3, 0.4, and 0.5 mol/L. After thorough mixing, the spectra were scanned in the range of 400 to 600 nm by a U-3900 spectrophotometer (Hitachi, Tokyo, Japan). The maximum absorption wavelengths at different NaOH concentrations were recorded. For blank control, distilled water was used to replace the BPS-2 samples, and the other steps specified above were repeated.

### 3.3. SEM and AFM Analyses

The surface morphology of the polysaccharides was studied by a Quanta 200 FEG SEM (FEI, Eindhoven, Netherlands). BPS-2 powder (3.0 mg) was fixed with double-sided adhesive carbon tape and then sprayed with gold under low vacuum conditions to make it conductive. SEM photographs were then obtained. Afterwards, the BPS-2 solution was heated to 85 °C for 10 min and filtered through a 0.22 μm aqueous-phase filter membrane to obtain the solution for the assay. A 2.0 μL aliquot of BPS-2 (0.01 μg/mL) solution was added dropwise on the surface of mica flakes and was then air-dried for Multimode 8-HR AFM (Bruker, Karlsruhe, Germany) measurements.

### 3.4. Thermogravimetric (TG) and Differential Scanning Calorimetry (DSC) Analyses

The TG performance of BPS-2 was tested by a Q500 TG analyzer (Waters, Milford, MA, USA). A 2.0 mg sample was placed in a ceramic crucible for TG analysis. The test conditions were as follows: temperature of 25 °C–400 °C and heating rate of 20 °C/min. The thermal properties of BPS-2 were tested by a Q200 DSC (Waters, Milford, MA, USA). A 2.0 mg sample was placed in a metal crucible for DSC analysis. 

### 3.5. X-Ray Diffraction (XRD) Analysis

The crystal structure of BPS-2 was assessed with a D2 PHASER XRD (Bruker Daltonics, Billerica, MA, USA). The detection conditions were a scanning angle range of 5° to 80° (2θ), scanning rate of 2θ = 0.1°/s, and data acquisition by continuous scanning.

### 3.6. Rheological Measurements

The apparent viscosity was measured by an MCR302 interfacial rheometer (Anton Paar, Graz, Austria). Different solutions containing 1–5% *w*/*v* BPS-2 (pH 7.0) and different pH solutions containing 3% *w*/*v* BPS-2 (pH 3–11) were used to measure the steady-state rheological properties of EPS. Using 0.6 mL of different assay solutions, detection was carried out at 25 °C using a 25 mm parallel splint, with the clamping distance set to 1.0 mm and the shear rate of 1–100 s^−1^.

### 3.7. Antioxidant Activity

The antioxidant activity was evaluated for DPPH, superoxide anion, hydroxyl, and ABTS radical scavenging as reported previously [47]. Briefly, the DPPH radical scavenging assay involved mixing BPS-2 with a DPPH solution, measuring the absorbance after standing in the dark, and calculating the scavenging rate. The superoxide anion radical scavenging assay utilized the generation of superoxide anion by the autoxidation of o-phenylthiol, and the scavenging rate was calculated by measuring the absorbance at a specific wavelength after the addition of BPS-2. The hydroxyl radical scavenging assay was performed by generating hydroxyl radicals through Fenton reaction, adding BPS-2 and salicylic acid to measure the absorbance, and calculating the scavenging rate. The ABTS radical scavenging assay was performed by reacting ABTS solution with potassium persulfate, diluting it, adding BPS-2 to measure the absorbance, and calculating the scavenging rate.

### 3.8. Anticancer Activity

Human colorectal cancer cell lines (SW480, HT29, and LoVo) and the human normal colonic epithelial cell line (HcoEpiC) were provided by the Shanghai Branch of the Chinese Academy of Sciences. The four cell lines were cultured in Dulbecco’s modified eagle medium (DMEM, HyClone; Logan, Utah, USA) containing 10% (*v*/*v*) fetal bovine serum with the addition of penicillin (100 μg/mL) and streptomycin (100 μg/mL). The cultures were incubated at 37 °C in a humidified 5% CO_2_ incubator. The effect of BPS-2 on the growth of four cell lines was assessed in vitro by MTT assay.

The cells were dispersed with 0.25% trypsin and adjusted to approximately 3 × 10^4^ cells/well in culture plates containing 100 μL of medium, and the cells were cultured for 24 h for apposition and were then supplied with 100 μL of fresh medium. Different concentrations (0–4000 μg/mL) of BPS-2 (100 μL) were added to the medium in each well. The cells were incubated for 24 h and microphotographs were taken. The medium containing BPS-2 was removed, and the attached cells were incubated in the medium containing 10 μL MTT solution for 3 h. After the change of solution, 200 μL DMSO was added, and the cells were shaken for 10 min at 25 °C. The absorbance at 490 nm was measured using a microplate reader.

### 3.9. Fermentation of EPS by Fecal Flora of Volunteers

The fresh stool samples were collected from two healthy volunteers and four UC volunteers. These donors all followed a normal Chinese Jiangsu diet, as detailed in Table 1. The study was approved by the Research Ethics Committee of Wuxi Second People’s Hospital, Wuxi, China (ethics number 2020002). All volunteers provided written informed consent agreeing to participate in the study. Feces were collected in sterile sealed containers and transported at low temperatures for use on the same day. The feces of each volunteer were diluted with sterile 0.1 M PBS at a ratio of 1:10 (*w*/*v*). The fecal homogenate was filtered through six layers of sterile gauze, and the filtrate was collected for inoculation. Basal fermentation medium (pH 5.8) contained the following (g/L): yeast extract 3.0, casein 2.0, tryptone 1.0, Tween 80 1.0, KCl 1.0, L-cysteine 0.5, NaCl 0.5, K_2_HPO_4_ 0.5, KH_2_PO_4_ 0.5, bile salt 0.4, CaCl_2_·6H_2_O 0.15, hemin 0.025, FeSO_4_·7H_2_O 0.005, and MgSO_4_·7H_2_O 0.01. Additionally, 1 mL of a vitamin mixture (g/L: pantothenic acid 10.0, nicotinamide 5.0, para-aminobenzoic acid 5.0, thiamine 4.0, D-biotin 2.0, menadione 1.0, and vitamin B12 0.5) was supplemented to the basal fermentation medium. To align with the study objectives, 5 g/L BPS-2 (treatment group) and 5 g/L starch (control group) were further incorporated.

The BGR was developed as an in vitro digestion model [48]. In this study, a colonic reactor of BGR was used (Figure S1). To the reactor, 180 mL of fermentation basal medium was added, heat-sterilized and cooled, and 20 mL of treated fecal homogenate was inoculated at a ratio of 1:10 (*v*/*v*). After inoculation, a temperature-controlled circulation pump was connected to maintain the internal temperature at 37 °C. The N_2_ was passed for 5 min to simulate an anaerobic environment in the intestine, maintaining the pH at 5.8–6.0. The fermentation medium was replenished with 30 mL of fresh medium every 8 h. The intestinal community was incubated for 24 h to reach a stable stage. This stage was set as the initial fermentation time, followed by feeding 30 mL of fermentation medium every 8 h. The experiment was terminated after 48 h of fermentation. Samples were collected and stored at −80 °C for testing.

### 3.10. 16S rRNA Gene Sequencing and Bioinformatics Analysis

The bacterial genomic DNA was extracted using a QIAamp DNA Stool Mini Kit (Qiagen, Hilden, Germany). The bacterial 16S rRNA gene was amplified using universal primers 341F (5′-CCTAYGGGRBGCASCAG-3′)/806R (5′-GGACTACHVGGGTWTCTAAT-3′). After sequencing, the 16S rRNA sequence data were bioinformatically analyzed according to the published methods [49].

### 3.11. Determination of SCFAs

The SCFAs were determined on a 7890A gas chromatograph (Agilent, Santa Clara, CA, USA). To 1 mL of fermentation supernatant, 10 μL of the internal standard 2-methylbutyric acid (1 mM), 250 μL of hydrochloric acid, and 1 mL of anhydrous ethyl ether were added, followed by vortexing for 5 min. Organic phase was collected, dehydrated with anhydrous sodium sulfate, and filtered through a 0.22 μm microporous membrane. The chromatographic column was an HP-INNOWax column (Restek, Bellefonte, PA, USA). The detector temperature was 250 °C, and the injector temperature was 220 °C. The oven temperature was started at 60 °C and increased to 190 °C after 4 min. Samples (5 μL) were injected with N_2_ carrier gas at a split ratio of 1:20. The concentration of SCFAs was calculated using an external standard method [50].

### 3.12. Statistical Analyses

The statistical analyses were performed in SPSS Statistics 19.0 (IBM, Armonk, New York, NY, USA). Data were expressed as mean ± standard deviation (S.D.). Statistically significant values were compared by ANOVA, and Dunnett’s test was used for multiple comparisons.

## 4. Conclusions

The study was based on the exploration of the multifunctional activity of BPS-2 (a derivative of GAGs synthesized by *B. thuringiensis* IX-01 through high-cell-density fermentation) and its potential applications. BPS-2 is a high-molecular-weight straight-chain polysaccharide with glucosamine and galactosamine as the backbone. Compared to other EPS reported in the literature, BPS-2 exhibits good functional properties in terms of thermal stability and rheological properties, and antioxidant and anticancer activity. In addition, it was confirmed that BPS-2 has a promising effect on the composition of the intestinal flora of UC patients while promoting the production of SCFAs. Thus, BPS-2 has potential as functional food to improve UC, but further in vivo experiments are needed to explore its mechanism of action in detail and to assess its safety as a food additive.

## Figures and Tables

**Table 1:** Summary of basic information about human fecal donors.

NO	1	2	3	4	5	6
Gender	female	male	female	male	female	male
Age	37	35	45	33	61	32
Disease History	none	none	UC	UC	UC	UC
Have you taken any antibiotics within a month?	none	none	none	none	none	none
Have you taken any medication for enteritis within half a month?	none	none	none	none	none	none
Have you taken any dietary supplements within a month?	none	none	none	none	none	none
Have you taken any probiotics or prebiotics within a month?	none	none	none	none	none	none

## Data Availability

Data is contained within the article and supplementary material.

## Supplementary Materials

The following supporting information can be downloaded at: https://www.mdpi.com/article/10.3390/ijms26041692/s1.

## Abbreviations

EPS, exopolysaccharides; UC, Ulcerative colitis; GAGs, Glycosaminoglycans; SEM, Scanning electron microscopy; AFM, Atomic force microscopy; TG, Thermogravimetric; DSC, Differential scanning calorimetry; XRD, X-ray diffraction; DPPH, 1,1-diphenyl-2-picrylhydrazyl; ABTS, 2,2′-azino-bis (3-ethylbenzothiazoline-6-sulfonic acid); IC_50_, half-maximal inhibitory concentrations; D-GalN, D-galactosamine; GlcN, glucosamine; CRC, colorectal cancer; MTT, 3-(4,5-dimethylthiazol-2-yl)-2,5-diphenyltetrazolium bromide; SCFAs, short-chain fatty acids; BGR, bionic gastrointestinal reactor; OTUs, Operational taxonomic units.
