# Supplementary figures and images for: In Vitro Assessment of Bacillus thuringiensis Exopolysaccharides and Their Effects on Gut Microbiota from Ulcerative Colitis In Vitro
Source: Int J Mol Sci. 2025 Feb 16;26(4):1692. doi: 10.3390/ijms26041692 (PMC11855630; doi:10.3390/ijms26041692)

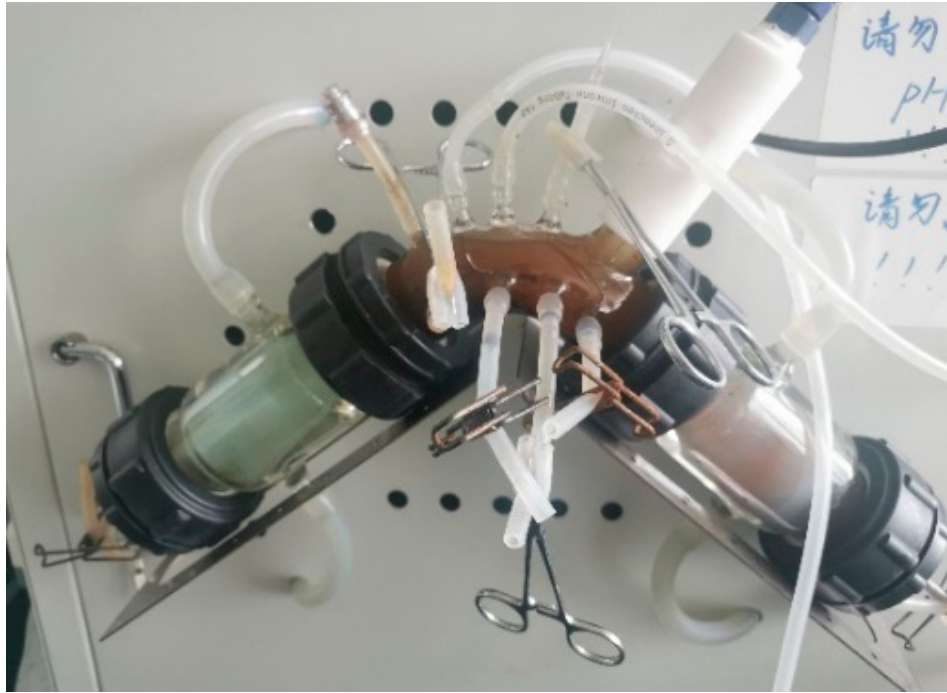

**Figure S1.** The colon reactors.

Supplement: Supplementary file 1 [file ijms-26-01692-s001.zip › ijms-3416869-supplementary.pdf]
